# Global regulation of heterochromatin spreading by Leo1

**DOI:** 10.1098/rsob.150045

**Published:** 2015-05-13

**Authors:** Laure Verrier, Francesca Taglini, Ramon R. Barrales, Shaun Webb, Takeshi Urano, Sigurd Braun, Elizabeth H. Bayne

**Affiliations:** 1 Institute of Cell Biology, University of Edinburgh, Edinburgh, UK; 2 Wellcome Trust Centre for Cell Biology, School of Biological Sciences, University of Edinburgh, Edinburgh, UK; 3 Butenandt Institute of Physiological Chemistry, Ludwig-Maximilians-Universität München, Munich, Germany; 4 Department of Biochemistry, Faculty of Medicine, Shimane University, Izumo, Japan

**Keywords:** heterochromatin, genome regulation, Leo1, epigenetics, fission yeast

## Abstract

Heterochromatin plays important roles in eukaryotic genome regulation. However, the repressive nature of heterochromatin combined with its propensity to self-propagate necessitates robust mechanisms to contain heterochromatin within defined boundaries and thus prevent silencing of expressed genes. Here we show that loss of the PAF complex (PAFc) component Leo1 compromises chromatin boundaries, resulting in invasion of heterochromatin into flanking euchromatin domains. Similar effects are seen upon deletion of other PAFc components, but not other factors with related functions in transcription-associated chromatin modification, indicating a specific role for PAFc in heterochromatin regulation. Loss of Leo1 results in reduced levels of H4K16 acetylation at boundary regions, while tethering of the H4K16 acetyltransferase Mst1 to boundary chromatin suppresses heterochromatin spreading in *leo1Δ* cells, suggesting that Leo1 antagonises heterochromatin spreading by promoting H4K16 acetylation. Our findings reveal a previously undescribed role for PAFc in regulating global heterochromatin distribution.

## 2. Introduction

The organization of eukaryotic genomes is fundamental to their integrity and regulation. DNA associates with histones and other proteins to form chromatin, and distinct patterns of post-translational histone modifications are associated with chromatin in different functional states [1]. Active chromatin domains, termed euchromatin, are characterized by high levels of histone acetylation and methylation of histone H3 at lysine 4 (H3K4me3), marks that confer an open chromatin conformation and facilitate transcription. By contrast, repressive chromatin, called heterochromatin, is characterized by low levels of histone acetylation and high levels of methylation at lysine 9 of histone H3 (H3K9me2) [2]. It has a compacted structure largely refractory to transcription, and is typically associated with transcriptional repression of underlying genes. While gene-rich regions are usually euchromatic, domains of heterochromatin such as those found at centromeres and telomeres play important roles in genome stability, contributing to centromere function, repression of recombination and maintenance of telomere integrity [2].

A key feature of heterochromatin is its inherent ability to ‘spread’ along the chromatin fibre via positive feedback mechanisms [3]. Methylation of H3K9 provides binding sites for the heterochromatin protein HP1, which recruits additional silencing factors and locks in the repressed state [4,5]. The H3K9 methyltransferase itself also binds methylated H3K9, as well as HP1, promoting further methylation of adjacent nucleosomes and hence spreading in *cis* [6–8]. This capacity to spread necessitates the existence of mechanisms that restrict heterochromatin to appropriate domains and prevent it encroaching into euchromatin, and potentially silencing essential genes. To some extent, expression levels of key silencing proteins such as HP1 may provide a general limitation on heterochromatin spreading [9,10]. In addition, the junctions between euchromatin and heterochromatin are often marked by specific boundary elements that provide barriers to heterochromatin spreading [11,12]. Several types of DNA sequence can serve as boundary elements, and diverse mechanisms appear to contribute to barrier activity; however, they typically function through either recruitment of enzymes responsible for depositing specific chromatin marks that antagonize heterochromatin formation [13,14], or tethering of the chromatin to the nuclear periphery to define physically distinct domains [15,16].

The fission yeast *Schizosaccharomyces pombe* has proved an important model organism for the study of heterochromatin assembly and regulation. Constitutive heterochromatin is found at centromeres, telomeres and the silent mating-type locus in fission yeast, and both heterochromatin structure and assembly pathways are broadly conserved from fission yeast to humans [2]. Assembly of heterochromatin in fission yeast has been shown to occur via a two-step process comprising nucleation and spreading, with several distinct mechanisms contributing to nucleation [17]. At telomeres and the silent mating-type locus, sequence-specific DNA binding proteins (Taz1 and Atf1/Pcr1, respectively) promote direct recruitment of factors required for heterochromatin establishment [18–21]. In addition, both these loci and the centromeric outer repeats contain related sequences that serve as nucleation centres for establishing heterochromatin via the RNA interference (RNAi) pathway. Non-coding transcripts generated from these regions are processed into siRNAs, which guide the RNAi effector complex RITS (comprising Ago1, Chp1 and Tas3) to homologous nascent transcripts [22–24]. Transcript-bound RITS mediates recruitment of the Clr4 complex (CLRC, comprising Clr4, Rik1, Raf1, Raf2 and Cul4) to cognate chromatin via the bridging protein Stc1, resulting in targeted H3K9 methylation [25]. Once established, the H3K9 methyl mark provides a binding site for chromodomain proteins, including both Clr4 and the HP1 protein Swi6 as well as RITS component Chp1; binding of these proteins contributes to a self-reinforcing loop that promotes propagation of heterochromatin beyond the sites of nucleation [4,8,26]. The activity of histone deacetylases including Sir2 and Clr3 is also important to generate the hypo-acetylated state and facilitate spreading of H3K9 methylation along the chromatin fibre [17,27,28].

**Figure 1.**
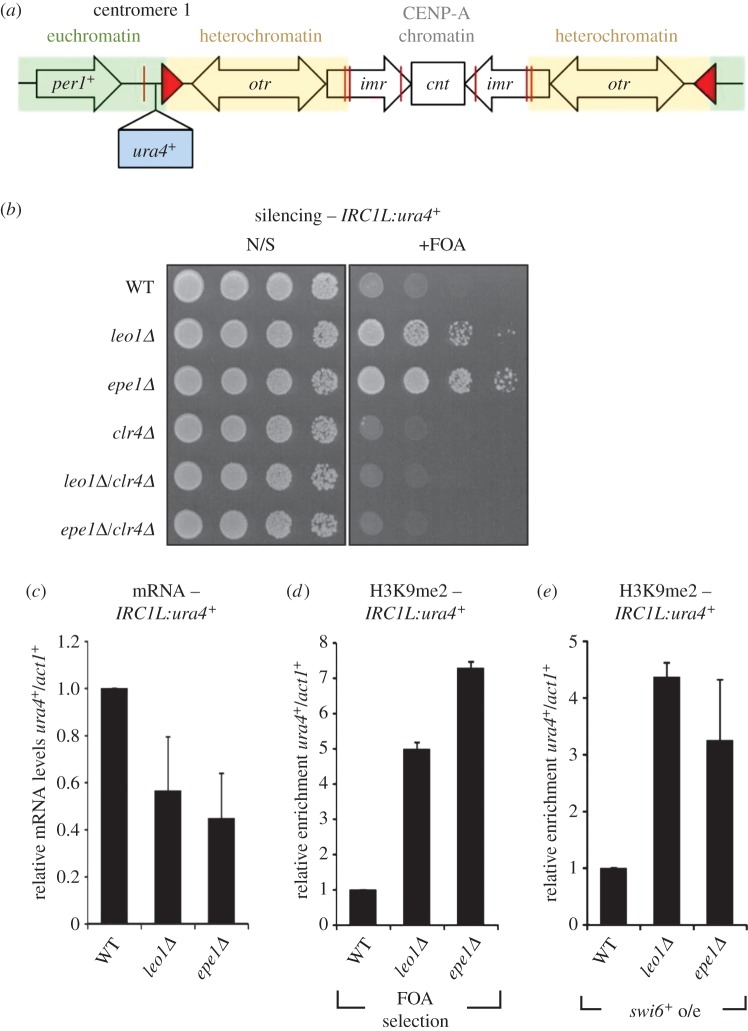
Leo1 is required to prevent spreading of heterochromatin across an *IRC* boundary. (*a*) Schematic showing the position of the *IRC1L:ura4^+^* insertion at centromere 1, relative to the outer repeats (*otr*), innermost repeats (*imr*), central domain (*cnt*), tRNA genes (red lines) and *IRC* elements (red triangles). (*b*) Assay for silencing at *IRC1L:ura4^+^*. Plates are non-selective (N/S) or supplemented with 5-FOA (+FOA); growth in the presence of 5-FOA indicates silencing of *ura4^+^.* (*c*) RT-qPCR analysis of *IRC1L:ura4^+^* transcript levels relative to a control transcript *act1^+^*, normalized to wild-type. (*d*,*e*) ChIP-qPCR analysis of H3K9me2 levels at the *IRC1L:ura4^+^*locus relative to the *act1^+^* gene, normalized to wild-type, in strains grown in the presence of 5-FOA (*d*), or overexpressing Swi6 (*e*). Data are averages of three biological replicates and error bars represent 1 s.d.

Although great strides have been made in understanding mechanisms promoting heterochromatin assembly in fission yeast, less is known about factors that regulate its spreading. The borders of heterochromatin domains at the silent mating-type locus and all three centromeres are characterized by sharp transitions in histone modification profiles that coincide with specific boundary elements [29]. At the mating-type locus, short inverted-repeat sequences termed IRs serve as boundary elements [29,30]. These sequences recruit the RNA polymerase III transcription factor TFIIIC, which associates with the nuclear periphery and is thought to physically partition the chromatin into distinct domains [16,31]. Fission yeast centromeres comprise a central core region characterized by a specialized form of chromatin containing the histone H3 variant CENP-A, flanked by outer repeat sequences that are assembled in heterochromatin (figure 1*a*). The junctions between centromeric heterochromatin and either CENP-A chromatin or euchromatin are frequently marked by clusters of tRNA genes. The precise mechanism by which tRNA genes generate boundary activity is unclear, but their boundary function requires both TFIIIC and RNAPIII, and may involve the formation of nucleosome-free regions refractory to heterochromatin spreading [32,33]. Loss of the histone demethylase Lsd1 is also associated with spreading of heterochromatin across both tRNA- and IR-delineated boundaries [34]. In addition, at centromeres 1 and 3 distinct inverted-repeat sequences termed *IRC*s serve as boundary elements between heterochromatin and flanking euchromatin. These do not bind TFIIIC, but are enriched for the JmjC domain-containing protein Epe1, a general negative regulator of heterochromatin [16,31,35]. In contrast to other heterochromatic regions, telomeric heterochromatin domains appear to lack defined boundary elements. In fact, two distinct chromatin transitions have been defined at telomeres: from heterochromatin to a specialized subtelomeric chromatin, and from subtelomeric chromatin to euchromatin [36]. The chromatin remodeller Fft3 is required to prevent invasion of euchromatin into subtelomeric chromatin, but how the transition between heterochromatin and subtelomeric chromatin is regulated is unknown [37].

Epe1 was identified as a factor required to prevent spreading of heterochromatin beyond normal boundaries in fission yeast, but has also been shown to regulate heterochromatin assembly independently of boundary elements [38,39]. In fact, Epe1 has been found to be recruited throughout heterochromatic domains via interaction with Swi6, but specifically depleted from all but the boundary regions due to Cul4-Ddb1 E3 ligase-dependent ubiquitination and degradation [35,40]. How Epe1 antagonises heterochromatin assembly is unclear, as although Epe1 bears structural similarity to histone demethylases, it does not display this activity *in vitro* [41,42]. However, a recent study uncovered a link between Epe1 and acetylation of histone H4 at lysine 16 (H4K16ac) at boundaries [43]. *IRC* boundaries in fission yeast are enriched for H4K16ac, and loss of this mark, for example by disruption of the acetyltransferase Mst1, impairs boundary function. Epe1 appears to help maintain H4K16ac at boundaries by recruiting the bromodomain protein Bdf2, which binds the H4K16ac mark and protects it from deacetylation by Sir2, thereby impeding heterochromatin spreading [43].

To uncover additional factors involved in chromatin boundary activity in fission yeast, we performed a genetic screen for mutants in which centromeric heterochromatin boundary function is impaired. We found that deletion of the PAF complex (PAFc) component Leo1 causes centromeric heterochromatin to spread across normal boundaries and invade euchromatin. Similar deregulation was seen upon deletion of other PAFc components, but not other factors linked to transcription elongation or transcription-coupled chromatin modification, indicating a specific role for this complex in heterochromatin regulation. Loss of Leo1 results in reduced levels of H4K16 acetylation at boundaries, and tethering of the H4K16 histone acetyltransferase Mst1 to chromatin can suppress heterochromatin spreading in the absence of Leo1, suggesting that Leo1 may inhibit propagation of heterochromatin domains by promoting H4K16 acetylation. Strikingly, genome-wide analyses revealed that loss of Leo1 results in expansion of heterochromatin domains at multiple genomic loci, particularly subtelomeres, indicating that Leo1 functions as a global regulator of heterochromatin spreading.

## 3. Results

### 3.1. Leo1 is required to prevent spreading of heterochromatin across an *IRC* boundary

To identify candidate negative regulators of heterochromatin *cis*-spreading, we performed a genome-wide screen for mutants exhibiting reduced expression of a *ura4^+^* reporter gene inserted immediately outside the *IRC* heterochromatin boundary element on the left side of centromere 1 (*IRC1L:ura4^+^*; figure 1*a*) [44]*.* In wild-type cells, this *ura4^+^* reporter gene is euchromatic and hence expressed; cells therefore grow poorly on media containing the counter-selective drug 5-FOA. In cells in which boundary function is impaired, such as those lacking the known heterochromatin regulator Epe1, spreading of heterochromatin onto the *ura4^+^* reporter represses its expression, leading to increased growth on 5-FOA (figure 1*b*). By screening a library of approximately 3000 strains bearing single non-essential gene deletions [45] (electronic supplementary material, figure S1), we identified *leo1^+^* as a novel gene required to prevent silencing of *IRC1L:ura4^+^*. To rule out any secondary effects of the genetic background, we generated a fresh *leo1Δ* deletion strain for further analysis. We confirmed that cells lacking Leo1 exhibit reduced expression of *IRC1L:ura4^+^*, as evidenced by enhanced resistance to 5-FOA, similar to cells lacking Epe1 (figure 1*b*). This was verified by RT-qPCR analysis, which revealed decreased levels of *ura4^+^* transcripts in both *leo1Δ* and *epe1Δ* cells (figure 1*c*). Interestingly, analysis of cells without the *IRC1L:ura4^+^* reporter gene revealed that loss of either Leo1 or Epe1 also results in a similar reduction in accumulation of transcripts from the endogenous *per1^+^* and *lys1^+^* genes located approximately 2 and 10 kb from the *IRC1L* element, respectively, indicating that increased silencing is not restricted to the reporter gene (electronic supplementary material, figure S2).

To determine whether silencing of *IRC1L:ura4^+^* in the absence of Leo1 is mediated by heterochromatin, we first tested whether it is dependent on the H3K9 methyltransferase Clr4. Deletion of Clr4 restored expression of *IRC1L:ura4^+^* in *leo1Δ* cells (figure 1*b*), confirming that Leo1 is required to prevent Clr4-dependent silencing beyond *IRC1L*. Because heterochromatin spreading is inherently stochastic, silencing of *IRC1L:ura4^+^* probably occurs only in a proportion of cells in a population at any one time. As observed previously in analyses of *epe1Δ* cells, this variability can make it difficult to detect changes in H3K9me2 levels at the population level by chromatin immunoprecipitation (ChIP) [35,43]. We therefore used two alternative strategies to increase the proportion of *IRC1L:ura4^+^*-silenced cells for ChIP analysis: (i) growth in the presence of 5-FOA, to select for cells undergoing *ura4^+^* silencing; or (ii) overexpression of the HP1 protein Swi6, which has been shown previously to lead to more robust silencing [16,29,43]. In combination with ChIP-qPCR, both strategies revealed increased levels of H3K9me2 at *IRC1L:ura4^+^* in *leo1Δ* cells and *epe1Δ* cells as compared with wild-type cells (figure 1*d,e*). This confirms that Leo1, like Epe1, is required to prevent spreading of centromeric heterochromatin into flanking euchromatin.

### 3.2. The Leo1-containing PAF complex has a specific role in restricting the spread of heterochromatin

**Figure 2.**
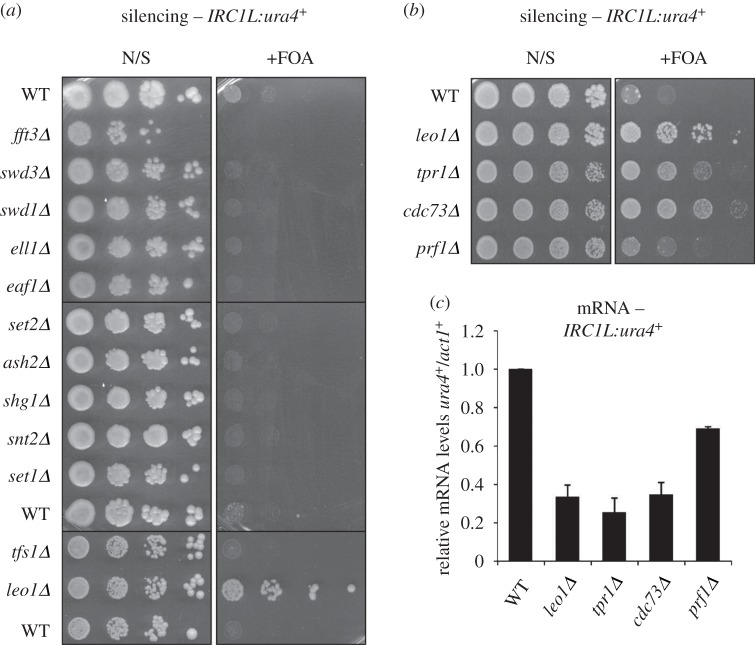
Loss of PAF complex components, but not other transcriptional regulators, results in silencing at *IRC1L:ura4^+^*. (*a*,*b*) Assay for silencing at *IRC1L:ura4^+^* in cells lacking factors involved in transcription elongation or transcription-coupled chromatin modification (*a*) or cells lacking PAFc components (*b*). Plates are non-selective (N/S) or supplemented with 5-FOA (+FOA). (*c*) RT-qPCR analysis of *IRC1L:ura4^+^* transcript levels relative to a control transcript *act1^+^*, normalized to wild-type. Data are averages of three biological replicates and error bars represent 1 s.d.

Leo1 is a component of PAFc, a conserved five-component complex comprising Paf1, Leo1, Tpr1(Ctr9), Cdc73 and Prf1(Rtf1) [46,47]. PAFc associates with RNA polymerase II (RNAPII) and contributes to the regulation of gene expression. In particular, PAFc is implicated in regulation of transcription elongation, in part via interactions with transcription elongation factors, but primarily due to multiple roles in promoting histone modifications associated with active transcription [46,48]. For example, PAFc facilitates trimethylation of H3K36 by promoting phosphorylation of RNAPII at Ser2, which in turn promotes recruitment of the methyltransferase Set2 [49,50]. PAFc also facilitates recruitment of enzymes that mediate monoubiquitination of histone H2B, which is necessary for Set1-dependent methylation of H3K4 [51–54]. Interestingly, in *S. cerevisiae*, the Leo1 subunit of PAFc appears to be dispensable for both H3K36 methylation and H2B monoubiquitination [49,51,52,55]. However, whether this is also the case in *S. pombe* is unknown. As PAFc is known to be involved in transcription regulation, we first investigated whether reduced expression of the *IRC1L:ura4^+^* reporter gene in *leo1Δ* cells could be the result of defective transcription. In addition to our earlier observation that the effect of *leo1^+^* deletion on *IRC1L:ura4^+^* expression is Clr4-dependent (figure 1*b*), we found that expression of *ura4^+^* inserted at another euchromatic locus is unaffected by loss of Leo1 (electronic supplementary material, figure S3). This argues against the possibility that loss of Leo1 simply impairs transcription of the *ura4^+^* reporter. Moreover, no other transcription-related mutants were recovered in the screen, as might be expected if the *leo1Δ* phenotype were a result of a general defect in transcription. To investigate this further, we retested *IRC1L:ura4^+^* expression in cells bearing single deletions of a range of non-essential factors involved in transcription elongation or transcription-coupled chromatin modification, including transcription elongation factors TFIIS (Tfs1), Ell1 and Eaf1 [56,57], SET1 H3K4 methyltransferase complex components (Set1, Swd1, Swd3, Shg1 and Ash2) [58], the H3K36 methyltransferase Set2 [59], and the Lid2 histone demethylase subunit Snt2 [58]. None of these mutants exhibited increased silencing of *IRC1L:ura4^+^* (figure 2*a*), confirming that the enhanced silencing observed in *leo1Δ* cells is specific, and unlikely to be attributable to a general transcription-related defect. Thus, fission yeast Leo1 may have a specific role in heterochromatin regulation that is independent of other functions of PAFc.

To investigate whether other components of PAFc function along with Leo1 in heterochromatin regulation, we tested whether single deletions of three other PAFc subunits also cause silencing of *IRC1L:ura4^+^*. Cells lacking Tpr1, Cdc73 or, to a lesser extent, Prf1 all exhibited reduced expression of *IRC1L:ura4^+^* as assessed by both silencing assays and qRT-PCR (figure 2*b*,*c*). That loss of Prf1 does not affect *IRC1L:ura4^+^* expression to the same extent as the other PAFc components is consistent with recent evidence suggesting that this protein may not be a core component of PAFc in fission yeast [47]. These findings therefore suggest that the increased silencing and H3K9 methylation seen at *IRC1L:ura4^+^* in *leo1Δ* cells probably reflects a specific role for PAFc as a whole in suppressing heterochromatin spread.

### 3.3. Leo1 antagonizes the spread of heterochromatin by facilitating H4K16 acetylation

**Figure 3.**
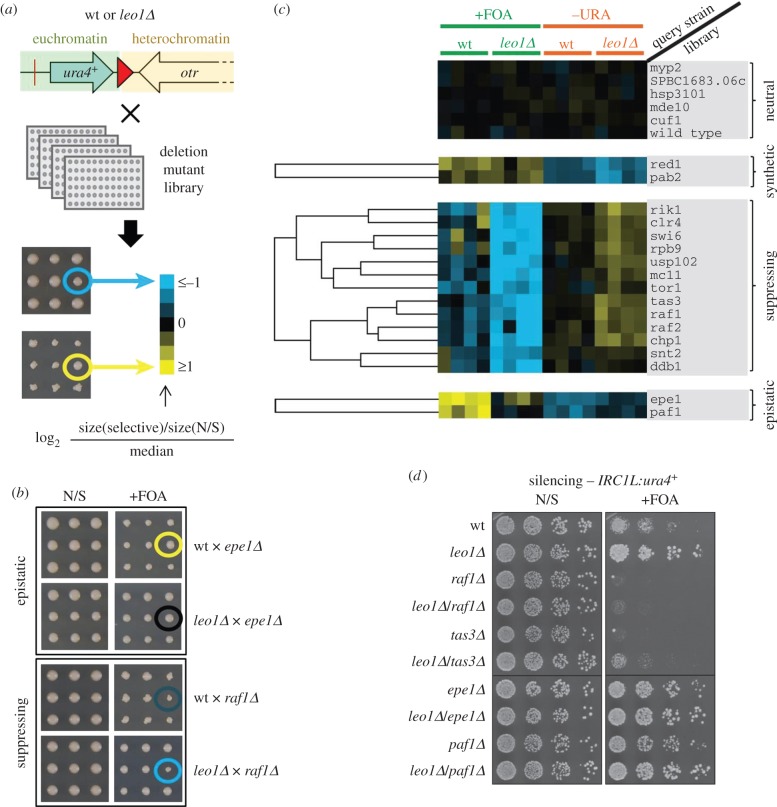
Identification of Leo1 genetic interactors. (*a*) Schematic of the SGA analysis. Wild-type or *leo1Δ* query strains (bearing the *IRC1L:ura4^+^* reporter and overexpressing *swi6^+^*) were crossed with the deletion library, and growth of the progeny was measured by colony size and represented as log_2_ values of the ratio of growth on selective media (+FOA or –URA) versus non-selective media, normalized to the median ratio (blue, small colonies; yellow, large colonies). (*b*) Examples of epistatic (upper panel) and suppressing (lower panel) interactions. The indicated mutants are circled. (*c*) Cluster analysis showing deletion mutants that suppress the *leo1Δ* phenotype at *IRC1L:ura4^+^*, or exhibit synthetic (aggravating) or epistatic interactions. The upper panel shows examples of mutants displaying no genetic interaction (neutral). Four replicates are shown for each experiment; blue indicates small colonies and yellow indicates large colonies. (*d*) *IRC1L:ura4^+^* silencing assay to validate SGA analysis results for the indicated strains.

To try to gain further insight into the function of Leo1 in heterochromatin regulation, we epitope-tagged Leo1 at the endogenous locus and performed affinity purification followed by liquid chromatography tandem mass spectrometry (LC-MS/MS) to identify interacting proteins. Paf1, Tpr1 and Cdc73 were all found to associate with Leo1, consistent with these proteins forming the core PAFc complex in fission yeast (electronic supplementary material, table S1). However, this analysis did not identify any additional Leo1-interacting proteins. As an alternative approach, we searched for mutants that interact genetically with *leo1Δ* by performing synthetic genetic array (SGA) analysis. Wild-type or *leo1Δ* query strains (each bearing the *IRC1L:ura4^+^* reporter, and overexpressing Swi6 to make silencing more robust) were crossed to the gene deletion library, and growth of the progeny on selective media (either lacking uracil or supplemented with 5-FOA) versus non-selective media was quantified, and the ratio compared with the median ratio (figure 3*a*,*b*). This analysis revealed that deletions of numerous factors with known roles in heterochromatin assembly and propagation suppress the *leo1Δ* heterochromatin-spreading phenotype, including Swi6, CLRC components Clr4, Rik1, Raf1 and Raf2, and RITS components Chp1 and Tas3 (figure 3*c*). The suppressive effects of a subset of these mutants were validated by silencing assays, which confirmed that the double mutants exhibit reduced *IRC1L:ura4^+^* silencing (reduced growth in the presence of 5-FOA) as compared with the *leo1Δ* single mutant (figure 3*d*). This finding is consistent with Leo1 functioning to antagonize the activity of proteins that promote heterochromatin formation. Conversely, the *leo1Δ* heterochromatin-spreading phenotype was found to be enhanced (synthetic interaction) by deletion of Red1 or Pab2 (figure 3*c*). As these factors are known to be required for facultative heterochromatin assembly at loci such as meiotic genes [60,61], this may reflect increased availability of silencing factors at centromeres due to their release from other sites. Notably, two mutants were found to be broadly epistatic to *leo1Δ*: deletions of the heterochromatin regulator Epe1, and the PAFc component Paf1 (figure 3*c*,*d*). While *epe1Δ* and *paf1Δ* single mutants exhibit similar phenotypes to *leo1Δ* cells, *paf1Δ/leo1Δ* and *epe1Δ/leo1Δ* double mutants exhibit little or no enhancement of the *leo1Δ* phenotype, indicating that these factors do not act synthetically/redundantly with Leo1, and may therefore function in the same pathway as Leo1. This supports our previous findings indicating that other PAFc components function along with Leo1 to suppress heterochromatin spreading, and additionally suggests that the similar phenotypes of cells lacking Epe1 or Leo1 may also reflect roles for these factors in the same heterochromatin regulation pathway.

**Figure 4.**
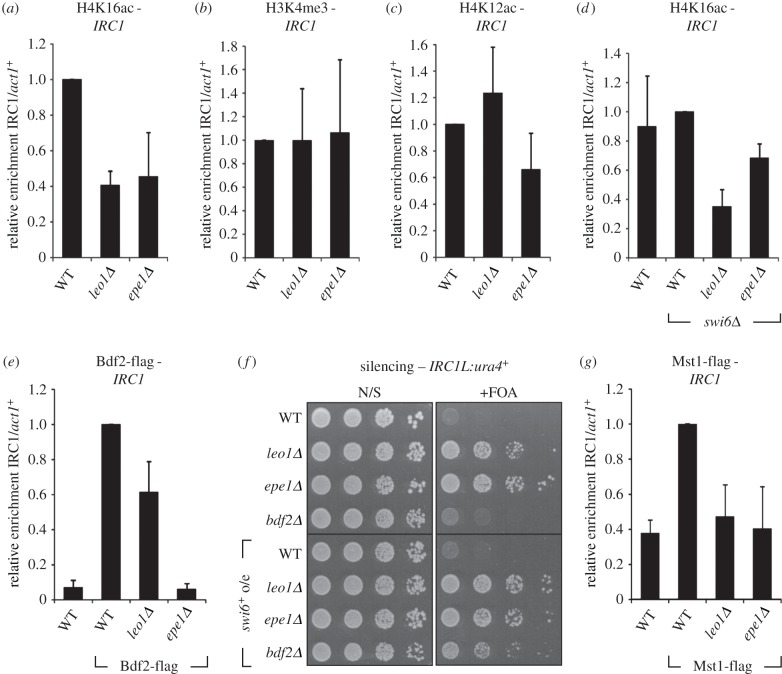
Loss of Leo1 results in reduced H4K16ac levels at the *IRC1* locus. (*a*) ChIP-qPCR analysis of H4K16ac levels at the endogenous *IRC1* element relative to the *act1^+^* gene, normalized to wild-type. (*b*,*c*) ChIP-qPCR analysis of levels of other transcription-associated chromatin marks, (*b*) H3K4me3 and (*c*) H4K12ac, at *IRC1* relative *act1^+^*, normalized to wild-type. (*d*) ChIP-qPCR analysis of H4K16ac levels at *IRC1* in strains lacking Swi6. (*e*) ChIP-qPCR analysis of Mst1-flag association with *IRC1* relative to *act1^+^*, normalized to wild-type. (*f*) Assay for silencing at *IRC1L:ura4^+^* in strains with or without Swi6 overexpression; plates are non-selective (N/S) or supplemented with 5-FOA (+FOA). (*g*) ChIP-qPCR analysis of Bdf2-flag association with *IRC1* relative to *act1^+^*, and normalized to wild-type. Data are averages of three biological replicates and error bars represent 1 s.d.

A simple explanation for the phenotypic relationship between *epe1Δ* and *leo1Δ* cells could be that loss of Leo1 affects either the expression of Epe1 or its localization to chromatin. However, q-RT-PCR and ChIP analyses revealed that deletion of Leo1 has no effect on either *epe1^+^* mRNA levels or association of Epe1 with the *IRC* boundary element, ruling out this possibility (electronic supplementary material, figure S4). It was recently reported that Epe1 contributes to boundary function at *IRC* elements by promoting high local levels of H4K16 acetylation, which inhibits heterochromatin spreading. H4K16 acetylation is mediated by Mst1, and protected from deacetylation by the bromodomain protein Bdf2, which is recruited via Epe1 [43]. Given that PAFc is known to be involved in recruitment of certain co-transcriptional chromatin modifiers, we hypothesized that it might also be important to facilitate H4K16 acetylation at boundaries. Consistent with this idea, ChIP analysis revealed reduced levels of H4K16ac at the endogenous *IRC* boundary element in *leo1Δ* cells, similar to what is seen in *epe1Δ* cells (figure 4*a*). By contrast, levels of two other chromatin marks associated with active transcription, H3K4me3 and H4K12ac, were largely unaffected at this locus (figure 4*b*,*c*); this argues that the loss of H4K16 acetylation at the *IRC* element is specific, rather than a reflection of a general loss of active chromatin marks as a consequence of reduced transcription. In principle, reduced H4K16 acetylation at the boundary could be either a cause or a consequence of heterochromatin spreading. However, deletion of Swi6, which is required for spreading of heterochromatin, partially rescued H4K16ac levels at the boundary in *epe1Δ* cells, but did not rescue H4K16ac levels in *leo1Δ* cells (figure 4*a*,*d*). This observation suggests that the decrease in H4K16ac in cells lacking Leo1 is independent of the propagation of H3K9me2, and is therefore likely to be a cause, rather than a consequence, of heterochromatin spreading.

The reduction in H4K16ac levels at the *IRC* boundary in *leo1Δ* cells could result from either reduced acetylation by Mst1, or increased deacetylation owing to decreased binding of Bdf2. To investigate whether loss of Leo1 affects binding of Bdf2 at the *IRC*, we analysed association of Bdf2 with *IRC* chromatin by ChIP. As reported previously, we found that association of Bdf2 with the *IRC* is abolished in *epe1Δ* cells; this is consistent with Epe1 being required for Bdf2 recruitment. By contrast, we observed only a partial reduction in Bdf2 levels at the *IRC* in *leo1Δ* cells (figure 4*e*). Given that loss of Leo1 also causes a reduction in H4K16ac at the *IRC* (figure 4*a*), it seems likely that this partial reduction in Bdf2 association reflects a reduction in available H4K16ac binding sites, rather than a specific role for Leo1 in Bdf2 recruitment. Moreover, a side-by-side comparison revealed that loss of either Leo1 or Epe1 results in much stronger silencing of *IRC1L:ura4^+^* than does loss of Bdf2 in both wild-type and *swi6^+^* over-expression backgrounds (figure 4*f*), indicating that spreading of heterochromatin in *leo1Δ* cells cannot be explained simply by a defect in recruitment or function of Bdf2. To assess whether loss of Leo1 might instead affect recruitment of the H4K16 acetyltransferase Mst1 to the *IRC*, we analysed association of Mst1 with *IRC* chromatin by ChIP. Levels of Mst1 at the *IRC* were found to be reduced in both leo1*Δ* and *epe1Δ* cells (figure 4*g*); this is consistent with the observed reduction in H4K16ac, and indicates that both Leo1 and Epe1 are important for efficient targeting of Mst1 to the *IRC*.

**Figure 5.**
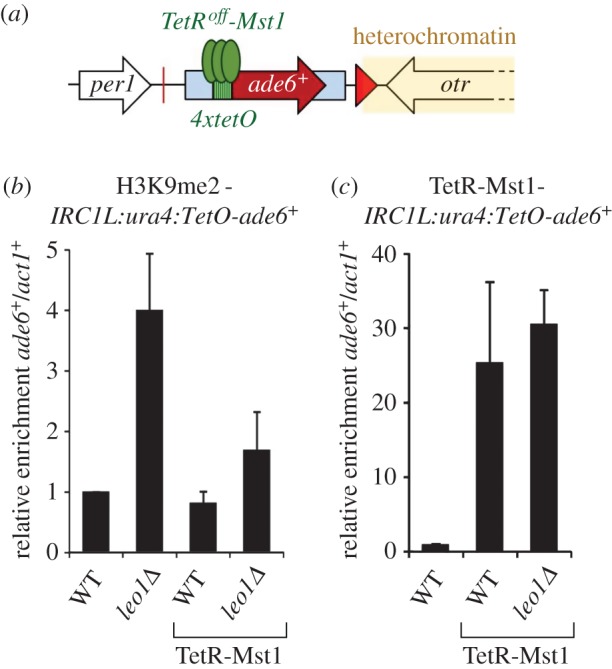
Tethering the histone acetyltransferase Mst1 is sufficient to suppress heterochromatin spreading in *leo1Δ* cells. (*a*) Schematic of the *IRC1L:ura4:TetO-ade6^+^* locus, which contains an *ade6^+^* reporter gene flanked by four *TetO* binding sites for recruitment of TetR-Mst1. (*b*) ChIP-qPCR analysis of H3K9me2 levels at the *IRC1L:ura4:TetO-ade6^+^* locus relative to the *act1^+^* gene, normalized to wild-type. (*c*) ChIP-qPCR analysis of TetR-Mst1 levels at the *IRC1L:ura4:TetO-ade6^+^* locus relative to the *act1^+^* gene. Data are averages of three biological replicates and error bars represent 1 s.d.

If heterochromatin spreading in the absence of Leo1 is indeed owing to a defect in recruitment of Mst1, then artificial tethering of Mst1 to the chromatin might be expected to restore boundary function in *leo1Δ* cells. To test this, we expressed Mst1 fused to a TetR^off^ DNA binding domain plus two FLAG tags (TetR-Mst1), and inserted four *TetO* binding sites alongside an *ade6^+^* reporter gene into the *IRC1L:ura4^+^* locus (*IRC1L:ura4:TetO-ade6^+^*; figure 5*a*). As expected, in the absence of tethered Mst1, deletion of Leo1 caused spreading of heterochromatin at the modified *IRC1L:ura4:TetO-ade6^+^* locus, resulting in increased levels of H3K9me2 on the *ade6^+^* reporter gene. Strikingly, however, tethering Mst1 to the chromatin largely abolished the increase in H3K9me2 in *leo1Δ* cells (figure 5*b*). ChIP analysis confirmed that the TetR-Mst1 fusion protein was enriched at the target locus (figure 5*c*). These analyses indicate that artificial recruitment of Mst1 can compensate for the loss of Leo1 in heterochromatin regulation, and therefore that Leo1 probably contributes to suppression of heterochromatin spreading by facilitating Mst1 recruitment and H4K16 acetylation. As we could not detect an interaction between Leo1 and Mst1 by co-immunoprecipitation combined with either mass spectrometry or Western blot (electronic supplementary material, table S1; some data not shown), Leo1-dependent recruitment of Mst1 may be mediated via another protein and/or chromatin mark.

### 3.4. Leo1 functions as a global regulator of heterochromatin independently of boundaries

**Figure 6.**
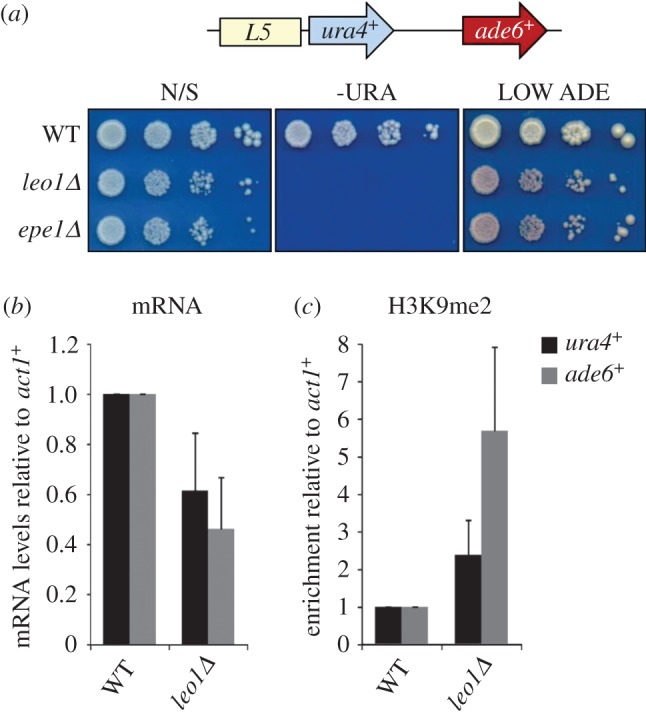
Leo1 regulates heterochromatin spreading independently of boundary elements. (*a*) Assay for silencing at the *ade6^+^:L5-ura4^+^* ectopic silencer locus. The schematic shows the arrangement of the locus comprising the L5 sequence (a 2.6 kb fragment of *otr* sequence) adjacent to a *ura4^+^* gene inserted at the euchromatic *ade6^+^* locus. Plates are non-selective (N/S), lacking uracil (−URA) or supplemented with limiting amounts adenine (LOW ADE). Silencing of *ura4^+^*results in loss of growth on –URA; silencing of *ade6^+^* results in red rather than white colonies on LOW ADE*.* (*b*) RT-qPCR analysis of *ura4^+^*and *ade6^+^*transcript levels relative to a control transcript *act1+*, normalized to wild-type. (*c*) ChIP-qPCR analysis of H3K9me2 levels at *ura4^+^*and *ade6^+^*relative to *act1^+^*, normalized to wild-type. Data are averages of three biological replicates and error bars represent 1 s.d.

Although certain chromatin regulators function only at specific boundary sequences, Epe1 has been found to be a global regulator of heterochromatin acting independently of boundaries [38–40]. To test whether this is also the case for Leo1, we assessed silencing at an ectopic heterochromatin locus where no known boundary elements are present. The ectopic locus consists of a 1.6 kb fragment of centromeric outer repeat sequence (L5) inserted alongside a *ura4^+^* reporter gene at the *ade6^+^* locus (*ade6^+^:L5-ura4^+^*; figure 6*a*). In wild-type cells, heterochromatin initiated on the repeat sequences causes partial silencing of the *ura4^+^* gene, but does not affect expression of the downstream *ade6^+^* gene [62]. Deletion of Epe1 causes increased silencing of both the *ura4^+^* and *ade6^+^* reporters, indicating spreading of heterochromatin [38]. Strikingly, cells lacking Leo1 also exhibit increased silencing of both *ura4^+^* (as evidenced by reduced growth on media lacking uracil) and *ade6^+^* (as evidenced by the appearance of red colonies; figure 6*a*). Reduced levels of *ura4^+^* and *ade6^+^* transcripts were detected in *leo1Δ* cells by qRT-PCR, and, moreover, ChIP analyses revealed elevated levels of H3K9me2 on both reporter genes in the absence of Leo1 (figure 6*b*,*c*; a greater fold change is seen at *ade6^+^* compared with *ura4^+^* as *ura4^+^* is already partially silenced in wild-type cells). Together these findings indicate that Leo1, like Epe1, can regulate heterochromatin spreading independently of any apparent boundary sequence.

**Figure 7.**
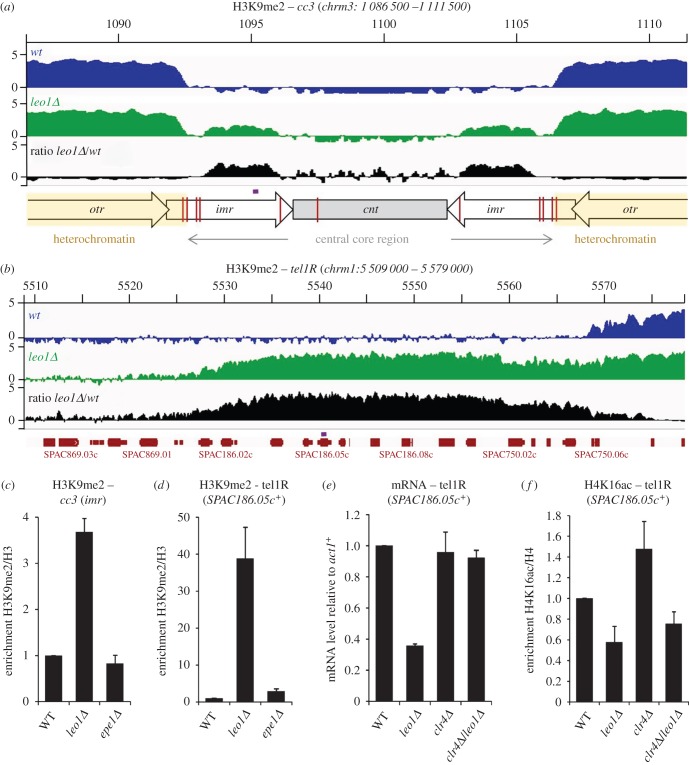
Leo1 functions as a global regulator of heterochromatin spreading. (*a*,*b*) Genome browser views showing ChIP-seq analysis of H3K9me2 levels in wild-type (blue) and *leo1Δ* (green) cells in log2 scale. *leo1Δ/wt* ratios are shown in black in linear scale. In each case, genome annotation is shown below; in the schematic in (*a*) red lines indicate the positions of relevant tRNA genes. The positions analysed by ChIP-qPCR are indicated in purple. (*c,d*) ChIP-qPCR analysis of H3K9me2 levels at the indicated loci relative to total H3, normalized to wild-type. (*e*) RT-qPCR analysis of *SPAC186.05c^+^* transcript levels relative to a control transcript *act1^+^*, normalized to wild-type. (*f*) ChIP-qPCR analysis of H4K16ac levels at the *SPAC186.05c^+^* locus relative to total H4, normalized to wild-type. ChIP-seq data represents the average of two biological replicates; other data are averages of three biological replicates and error bars represent 1 s.d.

As the experiments described above indicate that the role of Leo1 in heterochromatin regulation is not specific to *IRC* boundary elements, we investigated the effects of Leo1 deletion on H3K9me2 levels genome-wide by ChIP-seq analysis. This revealed pronounced changes in heterochromatin distribution at several sites in the genome. Within normal centromeric heterochromatin domains a small but uniform reduction in H3K9me2 levels was seen (figure 7*a*); this is consistent with a limited pool of silencing factors being redistributed to new domains. In addition to the documented spreading of centromeric heterochromatin outwards into flanking euchromatin, we also observed spreading of heterochromatin inwards into the central core of the centromeres, in particular at centromere 3 (*cc3*, figure 7*a*). This was validated by ChIP-qPCR analysis, which confirmed that *imr* repeat sequences that form part of the centromeric central core are associated with elevated levels of H3K9me2 in *leo1Δ* cells (figure 7*c*; note that normalization to histone H3 was performed to confirm that the observed increase in H3K9me2 does not simply reflect a change in incorporation of histone H3 in this region). Clusters of tRNA genes are thought to define the boundaries between heterochromatin and central core chromatin [32]; our observations indicate that Leo1 also plays a role in suppressing heterochromatin spreading at these sites. Interestingly, the strongest effects of Leo1 deletion were observed at the telomeres of chromosomes 1 and 2, which displayed substantial expansions of heterochromatin domains in comparison with wild-type cells (figure 7*b*; electronic supplementary material, figure S5). The right telomere of chromosome 1 (*tel1R*) displayed the greatest changes, with high levels of H3K9me2 extending an additional 40 kb away from the telomere (figure 7*b*). ChIP-qPCR analysis confirmed that H3K9me2 levels at *tel1R* are greatly increased in *leo1Δ* cells (figure 7*d*). In addition, qRT-PCR analysis showed that this rise in H3K9me2 levels is associated with a concomitant decrease in gene expression (figure 7*e*). The reduction in expression is dependent on Clr4, confirming that it is a consequence, rather than a cause, of heterochromatin spreading. To assess whether spreading of heterochromatin in this region is also linked to loss of H4K16ac, we analysed levels of H4K16 acetylation by ChIP-qPCR. As seen at centromeric (*IRC*) boundary elements, increased H3K9me2 at *tel1R* in *leo1Δ* cells is associated with a decrease in H4K16ac (figure 7*f*). Interestingly, deletion of Clr4 results in a small increase in H4K16ac; this suggests that low levels of heterochromatin may normally be present at this region even in wild-type cells. However, deletion of Leo1 in cells lacking Clr4 (and hence heterochromatin) still results in a reduction in H4K16ac levels, further supporting the idea that Leo1 antagonises the spread of heterochromatin by facilitating H4K16ac.

Our ChIP-qPCR analyses also revealed that accumulation of H3K9me2 at both *cc3* and *tel1R* is higher in *leo1Δ* cells than *epe1Δ* cells (figure 7*c*,*d*). Thus, Leo1 appears to play a greater role than Epe1 in regulating heterochromatin at these regions, with its activity being most critical at subtelomeres. Heterochromatic boundaries at telomeres do not appear to be defined by specific boundary sequences, but rather are suggested to result from a balance between active and repressive chromatin marks; the strong effects of Leo1 deletion at these sites are therefore consistent with Leo1/PAFc functioning as a global regulator of chromatin domain identity.

## 4. Discussion

Here, we uncover a previously undescribed role for the conserved PAFc in negative regulation of heterochromatin spreading. Our study focused on Leo1, which we identified in a genetic screen for factors required to prevent spreading of heterochromatin across a centromeric *IRC* boundary element. However, subsequent analyses revealed that deletion of other PAFc components results in similar heterochromatin spreading phenotypes, and that *leo1Δ* and *paf1Δ* mutants display epistatic interactions, suggesting that our observations on Leo1 reflect a role for PAFc as a whole in the regulation of heterochromatin spreading. Although relatively little studied in fission yeast, analyses in other organisms including budding yeast, flies and mammals have revealed conserved roles for PAFc in regulating transcription elongation and transcription-coupled chromatin modification [46,48]. While silencing of *IRC1L:ura4^+^* in *leo1Δ* cells could potentially have been due to defective transcription, our analyses indicate that this is unlikely to be the case; in particular, we found that deletion of Leo1 has no effect on expression of *ura4^+^* at a non-heterochromatic locus, while perturbing transcription via deletion of transcription elongation factors (Ell1, Eaf1 or Tfs1) or factors required for methylation of H3K4 (Set1/COMPASS components) or H3K36 (Set2) did not cause silencing of the *IRC1L:ura4^+^* reporter. In fact, this is consistent with evidence from budding yeast indicating that deletion of Leo1 has no discernible effect on either H3K4 or H3K36 methylation [49,51,52,55,63,64], and suggests that individual components of PAFc have distinct functions. In support of this, we note that single deletions of other PAFc components cause greater reductions in fission yeast cell viability than deletion of Leo1, suggesting that Leo1 is dispensable for one or more core functions of PAFc. This is consistent with the idea that Leo1 has little effect on transcription and may instead have a more specific function relating to heterochromatin regulation.

Little is known about the role of Leo1 in PAFc. However, our analyses revealed that at the *IRC* boundary element, deletion of Leo1 causes a specific reduction in H4K16 acetylation, uncovering a previously undescribed role for PAFc in regulation of this modification. Interestingly, association of the H4K16 acetyltransferase Mst1 with the boundary is also reduced in the absence of Leo1, and moreover, artificial tethering of Mst1 to the boundary largely suppresses the spreading of heterochromatin observed in *leo1Δ* cells. These observations suggest a model whereby Leo1/PAFc contributes to proper *IRC* boundary function by facilitating Mst1 recruitment and hence H4K16 acetylation. As recently described by Wang *et al*. [43], H4K16 acetylation at the boundary is protected from deacetylation by binding of Bdf2, creating a barrier to heterochromatin spreading. Precisely how Leo1/PAFc promotes recruitment of Mst1 is unclear, as we were unable to detect a physical interaction between Mst1 and Leo1 by co-immunoprecipitation combined with either mass spectrometry or Western blot (electronic supplementary material, table S1; some data not shown). However, as is the case for Set2, Leo1-dependent recruitment of Mst1 could be mediated via another protein and/or chromatin modification.

How PAFc is recruited to chromatin is not fully understood. PAFc subunits Rtf1/Prf1 and Cdc73 have both been shown to bind the phosphorylated form of the transcription factor Spt5, resulting in PAFc recruitment to transcribed genes [65–67]. In addition, Rtf1/Prf1 and Leo1 can bind RNA, and Leo1 is required for PAFc interaction with RNA and nucleosomes *in vitro* [68]. In the case of the *IRC* boundary, the *IRC* element is transcribed, giving rise to a non-coding RNA named *borderline* that is important for boundary function [69]. This raises the possibility that PAFc might be recruited to the *IRC* element via binding to the borderline RNA. However, given that PAFc is known to associate with active transcription units throughout the genome, and that the function of Leo1 in suppressing heterochromatin spreading is not restricted to *IRC* boundaries (see also below), it is unlikely that the borderline RNA itself is specifically required for PAFc recruitment. Rather, we suggest that the process of transcription may be sufficient to mediate recruitment of PAFc to *IRC* elements. PAFc has been found to associate with chromatin along the entire length of active genes [70,71], but to drive deposition of different chromatin marks in different contexts (e.g. H3K4me at the 5′ end of genes, and H3K36me in gene bodies) [46]. We therefore suggest that the function of Leo1/PAFc in facilitating H4K16ac at boundaries may be determined not through specific recruitment, but rather by chromatin context.

Side-by-side comparisons revealed that loss of either Leo1 or Epe1 has a greater impact on heterochromatin spreading at the *IRC*1L boundary than loss of Bdf2. This suggests that both Leo1 and Epe1 also have Bdf2-independent roles in heterochromatin regulation. It appears likely that these functions are linked, as at *IRC*1L the effects of deleting Leo1 or Epe1 are similar and largely epistatic to one another, and both proteins also affect spreading of heterochromatin at an ectopic locus with no known boundary element. Consistent with this, PAFc components Tpr1 and Cdc73 have also been reported to physically associate with Epe1 [43]. Interestingly, however, we identified other genomic loci, particularly telomeres, where loss of Leo1 has a much greater effect on heterochromatin spreading than does loss of Epe1 (see also below), indicating that in fact Leo1 plays an important role in global heterochromatin regulation that is related to, but distinct from, that of Epe1. Although the nature of the Bdf2-independent function of Epe1 remains unclear, phenotypic data support sequence-based predictions suggesting that Epe1 could function as a histone demethylase [41,42]. In the case of Leo1/PAFc, it is possible that this complex recruits one or more other chromatin modifiers in addition to Mst1 that contribute to heterochromatin regulation. In addition, a concurrent study has found evidence that PAFc also negatively regulates RNAi-mediated heterochromatin assembly via its role in promoting proper RNA 3′ end processing [72]. Given the importance of maintaining the identity of chromatin domains, it would not be surprising if interplay between multiple pathways contributes to heterochromatin regulation.

Genome-wide analyses revealed that loss of Leo1 results in a global redistribution of heterochromatin. In particular, we observed significant invasion of heterochromatin into the distinctive CENP-A chromatin that is found in the central core of the centromeres (in particular at *cc3*), as well as into the specialized subtelomeric chromatin that separates telomeric heterochromatin from euchromatin. It is interesting that the greatest degree of spreading in *leo1Δ* cells occurred at borders between heterochromatin and these unusual forms of chromatin, as it suggests that these transitions may be different and less well defined than heterochromatin–euchromatin boundaries. Indeed, at subtelomeres, where the greatest impact of Leo1 deletion was observed, defined boundary elements appear to be lacking. How heterochromatin is regulated at these loci is unclear, but it has been suggested that in the absence of boundary elements, transitions between distinct chromatin states can be determined dynamically by the balance of opposing chromatin modification activities [73]. Indeed, in budding yeast, which lacks H3K9me2, the borders of telomeric heterochromatin domains have been shown to depend on the balance between acetylation of H4K16 and Sir2-mediated deacetylation [74,75]. Our findings suggest that a similar mechanism operates in fission yeast, with Leo1/PAFc, a major regulator of chromatin modifications, playing an important role in the balance of repressive and active chromatin marks, particularly via regulation of H4K16 acetylation. Perturbations of this balance may have small effects at ‘fixed’ chromatin boundaries such as *IRC*, where the limits of heterochromatin are determined principally by defined sequence elements, but much greater effects at so-called ‘negotiable’ borders such as at telomeres.

By focusing on Leo1, we have uncovered a role for PAFc in heterochromatin regulation that appears distinct from other core functions of this complex in transcriptional regulation. Our findings shed new light on mechanisms governing the junctions between distinct chromatin domains in fission yeast, and provide novel insights into a previously uncharacterized role of Leo1/PAFc as a global regulator of chromatin domain identity. PAFc structure and function are broadly conserved throughout eukaryotes, and mutations in PAFc components have wide-ranging effects on development and disease [46]; it will therefore be important to investigate to what extent roles in regulation of H4K16 acetylation and heterochromatin spreading contribute to the impact of PAFc on gene regulation and genome integrity in higher eukaryotes.

## 5. Material and methods

### 5.1. Yeast strains and genetic analysis

Fission yeast strains used in this study are listed in electronic supplementary material, table S2. Standard procedures were used for growth and genetic manipulations. Genomic integrations including gene deletion and epitope-tagging were achieved by homologous recombination using PCR-based modules consisting of a resistance cassette flanked by sequences homologous to the target locus [76]. The *IRC1L*:*ura4*^+^ strain was constructed by insertion of the *ura4^+^* gene at the XhoI site just outside the *IRC* element on the left arm of chromosome 1 [44]. A nourseothricin (ClonNat) resistance cassette was inserted 4 kb upstream of the *IRC1L*:*ura4*^+^ locus to provide a means of selection for the reporter. The *IRC1L:ura4:TetO-ade6^+^* strain was constructed by amplifying a fragment consisting of four *TetO* binding sites adjacent to *ade6^+^* flanked by portions of the *ura4^+^* gene from plasmid *pW5/6-4xTetO-ade6^+^* as described previously [25], and inserting it into *IRC1L*:*ura4*^+^. The *ade6::L1(ura4+ade6+)* strain was described previously [62]. For serial dilution plating assays, 10-fold dilutions of cells were plated on the indicated media and grown at 32°C for 2–4 days.

### 5.2. Genetic screen of fission yeast deletion library

Screening was carried out using a near genome-wide haploid gene deletion library (v. 2.0) constructed and supplied by the Bioneer Corporation and the Korea Research Institute of Biotechnology and Bioscience [45]. Manipulations were performed using a Singer RoToR colony pinning robot, essentially as described previously [77]. First, the library was arrayed in 384 colony format, four colonies per deletion strain, on YES agar containing G418. The tester strain bearing the *IRC1L*:*ura4*^+^ reporter linked to a ClonNat resistance selectable marker was also arrayed in 384 colony format on YES agar containing ClonNat. Library and tester stain cells were then combined together on ME plates and incubated at 25°C for 3 days. The resulting cell/spore mixture was transferred onto selective media to select for haploid progeny bearing both the gene deletion and the *IRC1L:ura4^+^* reporter; these cells were then transferred to media supplemented with 5-FOA to screen for mutants exhibiting increased growth in the presence of 5-FOA, indicative of reduced expression of *IRC1L:ura4^+^*.

### 5.3. RNA analyses

Total RNA was extracted from 5 × 10^8^ cells in exponential growth phase using the RNAeasy Mini Kit (Qiagen) according to the manufacturer's instructions. After DNAse treatment for 1 h at 37°C (TURBO DNAseI, Ambion), 1 µg of total RNA was reverse transcribed using random hexamers (Roche) and Superscript III reverse transcriptase (Invitrogen) according to the manufacturer's instructions. cDNA was quantified by qPCR using LightCycler 480 SYBR Green (Roche) and primers listed in electronic supplementary material, table S3. In all cases, histograms represent three biological replicates and error bars represent 1 s.d.

### 5.4. Chromatin immunoprecipitation

ChIP experiments were performed essentially as described previously [25]. Briefly, 2.5 × 10^8^ cells per IP were fixed in 1% formaldehyde for 15 min at room temperature. Cells were lysed using a bead beater (Biospec products) and sonicated using a Bioruptor (Diagenode) for a total of 15 min (30 s on/30 s off). Immunoprecipitation was then performed overnight at 4°C using the following antibodies: anti-flag (2 µg per IP, FlagM2, Sigma), anti-H3K9me2 (1 µl per IP, 5.1.1 [78]), anti-H4K16ac (2 µg per IP, 39167, Active Motif), anti-H3K4me3 (1 µg per IP, 39 159, Active Motif), anti-H4K12ac (1 µg per IP, 39 165, Active Motif), anti-H3 (2 µl per IP, ab1791, Abcam) and anti-H4 (1.5 µl per IP, 05–858, Merck Millipore). Immunoprecipitated DNA was recovered using the Chelex-100 resin (BioRad), and quantified by qPCR using LightCycler 480 SYBR Green (Roche) and primers listed in electronic supplementary material, table S3. Relative enrichments were calculated as the ratio of product of interest to control product (*act1*^+^) in IP over input, or as percentage IP for modified histone over total histone. In all cases, histograms represent three biological replicates and error bars represent 1 s.d.

### 5.5. ChIP-seq analysis

ChIP experiments were performed as described above with the exception of DNA recovery. Following immunoprecipitation, cross-links were reversed using 1% SDS for 6 h at 65°C, and proteins removed by digestion with proteinase K (0.25 mg ml^−1^) for 2 h at 32°C. DNA was recovered using a Qiagen PCR purification kit, and libraries were constructed using 5 ng of input DNA or 16 to 20 ng of immunoprecipitated DNA. Briefly, after preparation of the DNA to generate blunt ends (Quick blunting kit, NEB; Klenow fragment synthesis, NEB), adaptors with internal barcodes were ligated using T4 DNA polymerase (NextFlex DNA barcodes-12, Bioo Scientific; Quick ligation kit, NEB). Libraries were then PCR amplified using Phusion High Fidelity DNA polymerase (NEB), according to the manufacturer's protocol; 15 and 12 cycles of amplification were performed for input and IP samples, respectively. AMPure XP magnetic beads (Beckman Coulter, Inc.) were used for purification and size exclusion between each step, according to the manufacturer's protocol. For multiplexed libraries, 50 nt paired end reads were sequenced on an Illumina Hiseq 2500 (Edinburgh Genomics, UK). Adapter removal and quality trimming were performed using Trimmomatic [79] and the processed sequences were aligned to the *S. pombe* ASM294 v. 2.22 genome assembly with Novoalign. Reads mapping to multiple locations were assigned a single random alignment to avoid double counting, and reads from replicate samples were merged and extended to match the paired end fragment size. Cross-sample normalization was achieved by scaling read depths to fragments per kilobase per million mapped reads (FPKM) using the deepTools bamCoverage tool. All data were converted to bigWig files for visualization in the integrative genome viewer (IGV [80]). Log2-fold changes of *leo1Δ* versus wild-type H3K9me2 signal were computed using deepTools bamCompare [81].

### 5.6. Synthetic genetic array analysis

The SGA screen was performed as described previously [82], with minor modifications. Briefly, query strains (wild-type and *leo1*Δ*,* each bearing the *IRC1L*:*ura4*^+^ reporter and overexpressing *swi6^+^*) and deletion mutants (*Bioneer* haploid deletion mutant library, v. 3.0) were arrayed in 384-format and mated on SPAS plates. Two independent mating rounds were performed for each query strain. After mating, plates were incubated at 42°C for 3 days to eliminate unmated haploid and non-sporulated diploid cells. Germination of spores was done on YES containing hygromycin B or G418 for *leo1*Δ and wild-type crossed strains, respectively. During this step, the array was converted into 768-format, resulting in four replicates (two copies for each mating). Cells were then transferred onto EMM plates lacking leucine to select for the *swi6^+^* overexpression cassette, and then onto YES plates containing G418 and ClonNat to select for the library gene deletion and the *IRC1L*:*ura4*^+^ reporter, respectively. Where necessary, plates containing hygromycin B were used to select for *leo1*Δ cells. Finally, cells were transferred onto EMM, EMM containing 5-fluoroorotic acid (FOA) (1 mg ml^−1^) and EMM lacking uracil. During this step, the 768 arrays were split into two copies in 384-format. All these steps were performed using the RoToR HDA colony pinning robot (Singer). All steps were performed at 30°C and all antibiotics were used at 100 µg ml^−1^. For growth analysis, digital pictures of the plates were taken after 2 days of growth, and sizes of individual yeast colonies were calculated using HT-colony-grid-analyzer software [83]. For all individual mutants, the ratio between growth on selective and non-selective media was determined, and then normalized to the median ratio of the respective 384-plate. Log2 values were used for hierarchical clustering analysis and visualization using Cluster v. 3.0 and TreeView software, respectively.

## Supplementary Material

Supplementary Information: Verrier et al

## Data accessibility

ChIP-seq data have been submitted to the Gene Expression Omnibus under accession no. GSE61688.
