# Supplementary material for: Global regulation of heterochromatin spreading by Leo1
Source: Open Biol. 2015 May 13;5(5):150045. doi: 10.1098/rsob.150045 (PMC4450266; doi:10.1098/rsob.150045)
Supplement: Supplementary Information: Verrier et al [file rsob150045supp1.pdf]

Global Regulation of Heterochromatin Spreading by Leo1

Laure Verrier[1,5], Francesca Taglini[1¶], Ramon R. Barrales[2¶], Shaun Webb[3], Takeshi Urano[4], Sigurd Braun[2], Elizabeth H. Bayne[1*]

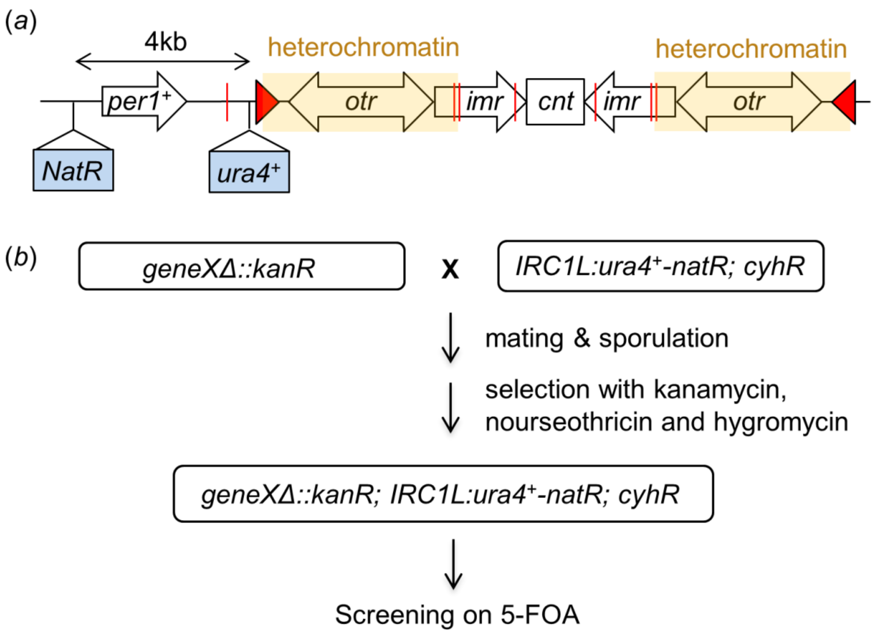

Figure S1. Genetic screen for mutants defective in heterochromatin boundary function. (*a*) Schematic diagram of insertions at centromere 1 in the tester strain used for the screen, showing the centromeric outer repeats (*otr*), innermost repeats (*imr*) central core (*cnt*), tRNA genes (red lines) and IRC elements (red triangles). $ura4^+$ inserted just outside the IRC element on the left side of the centromere (*IRC1L:*$ura4^+$) serves as a reporter gene to monitor heterochromatin spreading. A nourseothricin resistance cassette (NatR) inserted upstream of the $per1^+$ locus, approximately 4kb from the $ura4^+$ gene, allows selection for the reporter during crossing. The strain also contains the P56Q allele of the ribosomal protein gene $rpl42^+$, which confers recessive resistance cyclohexamide (cyhR) thereby providing a means of selecting against diploids. (*b*) Strategy to introduce the *IRC1L:*$ura4^+$ reporter into the fission yeast deletion library. Deletions in the fission yeast knockout strains are marked with a kanamycin resistance cassette (kanR); high throughput crossing of the tester strain to the deletion library followed by plating on selective media allowed selection for haploid progeny bearing both the *IRC1L:*$ura4^+$ reporter and a single gene deletion. These cells were then transferred to media supplemented with 5-FOA to screen for mutants exhibiting increased growth in the presence of 5-FOA, indicative of reduced expression of *IRC1L:*$ura4^+$.

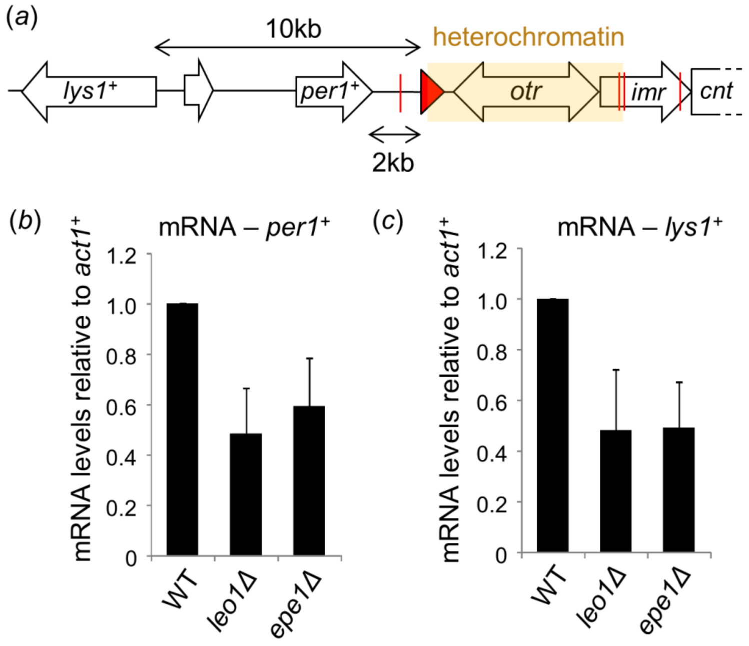

Figure S2. Deletion of Leo1 also results in reduced expression of endogenous genes close to the IRC1L boundary. (*a*) Schematic illustrating the positions of the *per1*⁺ and *lys1*⁺ genes close to the left side of centromere 1 (*otr*, outer repeats; *imr*, innermost repeats; *cnt*, central core). tRNA genes are indicated by red lines and the IRC1L element is shown as a red triangle. (*b*) and (*c*) RT-qPCR analysis of *per1*⁺ and *lys1*⁺ transcript levels relative to a control transcript *act1*⁺, normalized to wild-type. Data are averages of 3 biological replicates and error bars represent one SD.

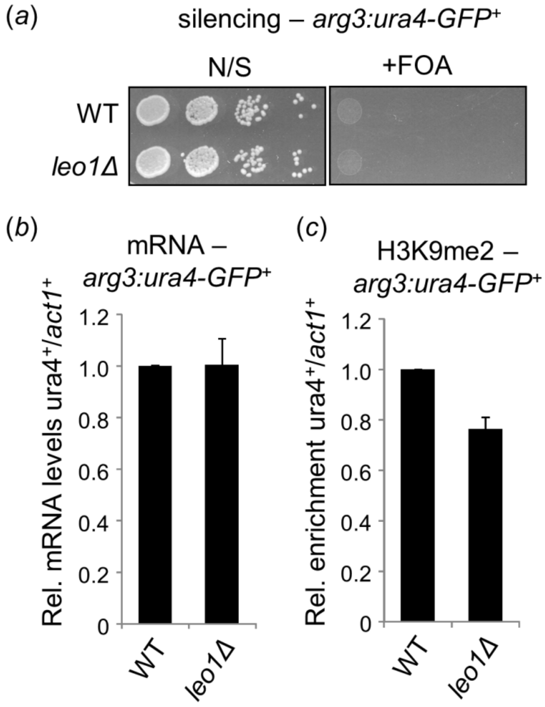

Figure S3. Deletion of Leo1 does not affect expression of *ura4*$^{+}$ at another euchromatic locus. (*a*) Assay for silencing *of ura4*$^{+}$ inserted at the euchromatic *arg3*$^{+}$ locus (*arg3::ura4*$^{+}$*-GFP*). Plates are non-selective (N/S) or supplemented with 5-FOA (+FOA). (*b*) RT-qPCR analysis of *arg3::ura4*$^{+}$*-GFP* transcript levels relative to a control transcript *act1*+, normalized to wild-type. (*c*) ChIP-qPCR analysis of H3K9me2 levels at the *arg3::ura4*$^{+}$*-GFP* locus relative to the *act1*+ gene, normalised to wild-type. Data are averages of 3 biological replicates and error bars represent one SD.

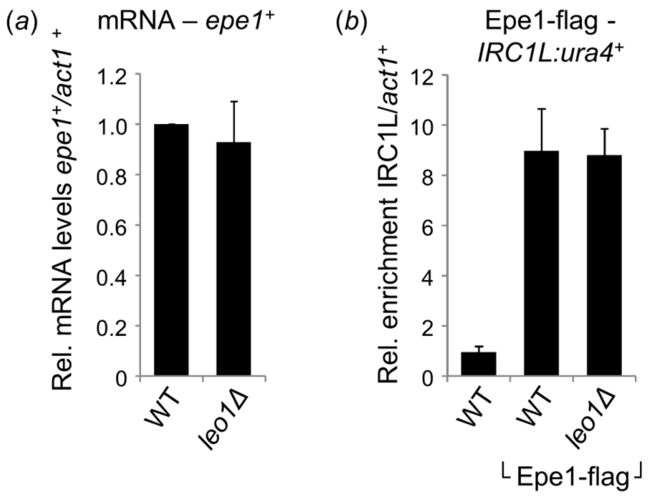

Figure S4. Deletion of Leo1 does not affect expression or localisation of Epe1. (*a*) RT-qPCR analysis of *epe1*$^{+}$ transcript levels relative to a control transcript *act1*$^{+}$, normalized to wild-type. (*b*) ChIP-qPCR analysis of Epe1-flag association with *IRC1L* relative to *act1*$^{+}$*,* normalised to wild-type. Data are averages of 3 biological replicates and error bars represent one SD.

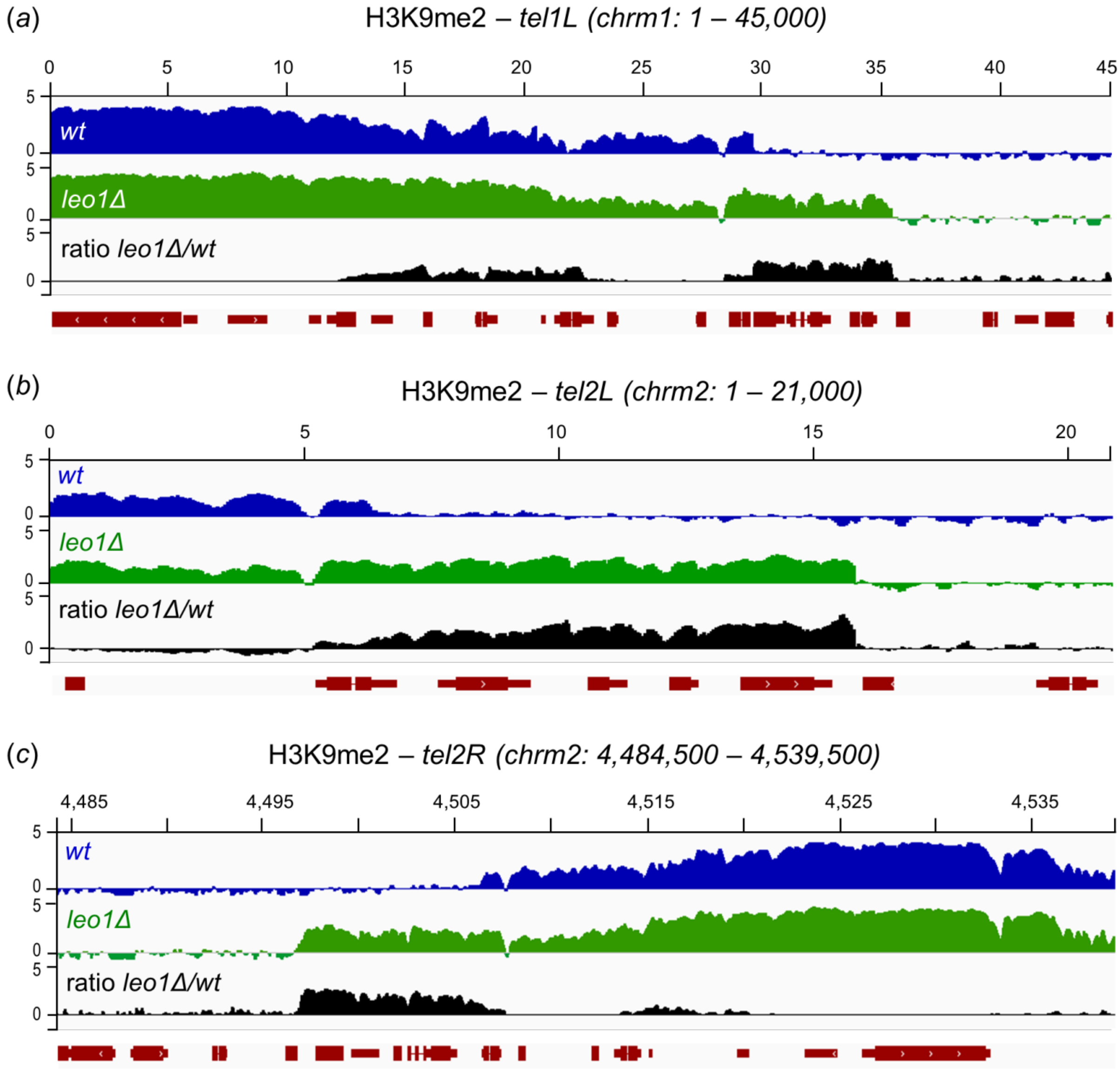

Figure S5. Deletion of Leo1 causes spreading of heterochromatin at telomeres.

Genome browser views showing ChIP-seq analysis of H3K9me levels at tel1L (*a*), tel 2L (*b*) and tel2R (*c*) in wild-type (blue) and *leo1Δ* (green) cells in log2 scale. *leo1Δ/wt* ratios are shown in black in linear scale and genome annotations are shown below. Data represents the average of two biological replicates.

Table S1. Proteins associating with Leo1. List of all proteins for which two or more peptides were identified in two independent affinity purifications of Leo1-FLAG, and no peptides were identified in control purifications. The peptide count represents the average number of unique peptides identified in the two purifications. The bait protein is highlighted in bold.

Protein	Peptide count	Mol weight (kDa)
Tpr1	28.5	119.1
**Leo1**	14	48.7
Cdc73	13	42.6
Paf1	9.5	51.3

Table S2. List of strains used in this study.

Strain	Genotype
EHB742	*h- IRC1L(Xho1):ura4$^+$ per1:NatR cycR ade6-210 leu1-32 ura4-D18*
EHB1397	*h+ leo1Δ::KanR IRC1L(Xho1):ura4$^+$ per1:NatR cycR? ade6-210 leu1-32 ura4-D18*
EHB1398	*h+ epe1Δ::KanR IRC1L(Xho1):ura4$^+$ per1:NatR cycR? ade6-210 leu1-32 ura4-D18*
EHB2586	*clr4Δ::LEU2$^+$ IRC1L(Xho1):ura4$^+$ per1:NatR ade6-210 leu1-32 ura4-D18*
EHB2588	*leo1Δ::KanR clr4::LEU2$^+$ IRC1L(Xho1):ura4$^+$ per1:NatR ade6-210 leu1-32 ura4-D18*
EHB2590	*epe1Δ::KanR clr4::LEU2$^+$ IRC1L(Xho1):ura4$^+$ per1:NatR ade6-210 leu1-32 ura4-D18*
EHB1550	*h- ars1:pREP81xGFP-Swi6-LEU2$^+$ IRC1L(Xho1):ura4$^+$ $^+$ per1:NatR ade6-210 leu1-32 ura4-D18*
EHB2414	*h- leo1Δ::KanR ars1:pREP81xGFP-Swi6-LEU2$^+$ IRC1L(Xho1):ura4$^+$ $^+$ per1:NatR ade6-210 leu1-32 ura4-D18*
EHB2416	*h- epe1Δ::KanR ars1:pREP81xGFP-Swi6-LEU2$^+$ IRC1L(Xho1):ura4$^+$ per1:NatR ade6-210 leu1-32 ura4-D18*
EHB2381	*fft3Δ::KanR IRC1L(Xho1):ura4$^+$ per1:NatR ade6-210/216 leu1-32 ura4-D18*
EHB2383	*swd3Δ:: IRC1L(Xho1):ura4$^+$ $^+$ per1:NatR ade6-210/216 leu1-32 ura4-D18*
EHB2385	*swd1Δ::KanR IRC1L(Xho1):ura4$^+$ per1:NatR ade6-210/216 leu1-32 ura4-D18*
EHB2387	*ell1Δ::KanR IRC1L(Xho1):ura4$^+$ per1:NatR ade6-210/216 leu1-32 ura4-D18*
EHB2389	*eafΔ::KanR IRC1L(Xho1):ura4$^+$ per1:NatR ade6-210/216 leu1-32 ura4-D18*
EHB2462	*set2Δ::KanR IRC1L(Xho1):ura4$^+$ per1:NatR ade6-210/216 leu1-32 ura4-D18*
EHB2395	*ash2Δ::KanR IRC1L(Xho1):ura4$^+$ per1:NatR ade6-210/216 leu1-32 ura4-D18*
EHB2397	*sgh1Δ::KanR IRC1L(Xho1):ura4$^+$ per1:NatR ade6-210/216 leu1-32 ura4-D18*
EHB2399	*snt2Δ::KanR IRC1L(Xho1):ura4$^+$ per1:NatR ade6-210/216 leu1-32 ura4-D18*
EHB2401	*set1Δ::KanR IRC1L(Xho1):ura4$^+$ per1:NatR ade6-210/216 leu1-32 ura4-D18*
EHB2554	*tpr1Δ::KanR IRC1L(Xho1):ura4$^+$ per1:NatR ade6-210/216 leu1-32 ura4-D18*
EHB2556	*cdc73Δ::KanR IRC1L(Xho1):ura4$^+$ per1:NatR ade6-210/216 leu1-32 ura4-D18*
EHB2517	*rtf1Δ::KanR IRC1L(Xho1):ura4$^+$ per1:NatR ade6-210/216 leu1-32 ura4-D18*
EHB2566	*h- leo1Δ::HygR ars1:pREP81xGFP-Swi6-LEU2 IRC1L(Xho1):ura4$^+$ per1:NatR ade6-210 leu1-32 ura4-D18*
EHB2568	*h- epe1Δ::HygR ars1:pREP81xGFP-Swi6-LEU2 IRC1L(Xho1):ura4$^+$ per1:NatR ade6-210 leu1-32 ura4-D18*
EHB9	*h+ ade6-210 arg3-D4 his3-D1 leu1-32 ura4-D18*

EHB1401	*h+ leo1Δ::KanR ade6-210 arg3-D4 his3-D1 leu1-32 ura4-D18*
EHB1402	*h+ epe1Δ::KanR ade6-210 arg3-D4 his3-D1 leu1-32 ura4-D18*
EHB106	*h- swi6Δ::NatR ade6-210 leu1-32 arg3D4 his3D1 ura4-D18*
EHB2475	*leo1Δ::KanR swi6Δ::NatR ade6-210 leu1-32 arg3D4 his3D ura4-D18*
EHB2477	*epe1Δ::KanR swi6Δ::NatR ade6-210 leu1-32 arg3D4 his3D ura4-D18*
EHB2449	*h- Mst1-3xflag-NatR ade6-210 arg3D4 his3D1 leu1-32 ura4-D18*
EHB2451	*leo1Δ::KanR Mst1-3xflag-NatR ade6-210 arg3D4 his3D1 leu1-32 ura4-D18*
EHB2453	*epe1Δ::KanR Mst1-3xflag-NatR ade6-210 arg3D4 his3D1 leu1-32 ura4-D18*
EHB2438	*h+ Bdf2-3xflag-NatR ade6-210 arg3D4 his3D1 leu1-32 ura4-D18*
EHB2447	*leo1Δ::KanR Bdf2-3xflag-NatR ade6-210 arg3D4 his3D1 leu1-32 ura4-D18*
EHB2445	*epe1Δ::KanR Bdf2-3xflag-NatR ade6-210 arg3D4 his3D1 leu1-32 ura4-D18*
EHB2527	*bdf2Δ::KanR IRC1L(Xho1):ura4^{+} per1:NatR ade6-210 leu1-32 ura4-D18*
EHB2549	*bdf2Δ::KanR ars1:pREP81xGFP-Swi6-LEU2^{+} IRC1L(Xho1):ura4^{+} per1:NatR ade6-210 leu1-32 ura4-D18*
EHB2173	*h+ IRC1L(Xho1):ura4:4xTetO-ade6^{+} per1:NatR ade6-DN/N leu1-32 ura4-D18*
EHB2266	*h+ leo1Δ::HygR IRC1L(Xho1):ura4:4xTetO-ade6^{+} per1:NatR ade6-DN/N leu1-32 ura4-D18*
EHB2273	*nmt81-TetRoff-2xflag-Mst1-leu1^{+} IRC1L(Xho1):ura4:4xTetO-ade6^{+} per1:NatR ade6-DN/N leu1-32 ura4-D18*
EHB2274	*leo1Δ::HygR nmt81-TetRoff-2xflag-Mst1-leu1^{+} IRC1L(Xho1):ura4:4xTetO-ade6^{+} per1:NatR ade6-DN/N leu1-32 ura4-D18*
EHB1460	*h- ade6^{+}:L5-ura4^{+} arg3D4 his3D1 leu1-32 ura4-DSE*
EHB2403	*leo1Δ::KanR ade6^{+}:L5-ura4^{+} arg3-D4 his3-D1 leu1-32 ura4-D18/DSE*
EHB2404	*epe1Δ::KanR ade6^{+}:L5-ura4^{+} arg3-D4 his3-D1 leu1-32 ura4-D18/DSE*
EHB1759	*h- arg3:ura4-GFP^{+} ade6-GFP^{+}-NatR arg11::HygR cycR leu1-32 ura4-D18*
EHB1424	*leo1Δ::KanR arg3:ura4-GFP^{+} ade6-GFP^{+}-NatR arg11::HygR cycR leu1-32 ura4-D18*
EHB1000	*h- ade6-M210 leu1-32 ura4-D18 epe1:CBP-FLAG-NatR*
EHB2705	*leo1Δ::KanR ade6-M210 leu1-32 ura4-D18 epe1:CBP-FLAG-NatR*

Table S3: List of qPCR primers used in this study.

Name	Sequence
q_ura4_F	CGTGGTCTCTTGCTTTTGG
q_ura4_R	GTAGTCGCTTTGAAGGTTAGG
q_act1_F	GGTTTCGCTGGAGATGATG
q_act1_R	ATACCACGCTTGCTTTGAG
q_IRC_F	CTCAATCCGTGGACGTATCA
q_IRC_R	CATCCTTTGCGTGAATCAGA
q_ade6_F	ATGCTTATCCTACAACTGAGACC
q_ade6_R	TGAATTGAGAAGGGAAGACGAG
q_tel1R_F	CAAAGGAGAAGGTGCTTGATG
q_tel1R_R	GCTACCATTGTACACAGCATATG
q_cc3_F	GCACGTTCTTACTGGTAGTTG
q_cc3_R	CTGCAGACATGGGAAAACAG
q_lys1_F	CCCAGCTATGGGTCAACTGT
q_lys1_R	CGGCTGGAATGACATCTTTT
q_per1_F	GCGGTGGTGTTCCTACTGAT
q_per1_R	GCACGAGGGAGAGACTTTTG
